# Dietary perturbations alter the ecological significance of ingested *Lactobacillus plantarum* in the digestive tract

**DOI:** 10.1038/s41598-017-07428-w

**Published:** 2017-08-04

**Authors:** Xiaochen Yin, Bokyung Lee, Jose Zaragoza, Maria L. Marco

**Affiliations:** 1 Department of Food Science and Technology, University of California, Davis, USA; 2 Department of Plant Pathology, Univeristy of California, Davis, CA USA; 3 Center for Comparative Medicine, Department of Anatomy, Physiology and Cell Biology, School of Veterinary Medicine, University of California, Davis, CA USA; 4 Bayer Crop Science, West Sacramento, CA USA

## Abstract

Host diet is a major determinant of the composition and function of the intestinal microbiome. Less understood is the importance of diet on ingested strains with probiotic significance. We investigated the population dynamics of exogenous *Lactobacillus plantarum* and its interactions with intestinal bacteria in mice undergoing switches between high-fat, high-sugar (HFHSD) and low-fat, plant-polysaccharide rich (LFPPD) diets. The survival and persistence of ingested *L*. *plantarum* WCFS1 was significantly improved during mouse consumption of HFHSD and was negatively associated with the numbers of indigenous *Lactobacillus* species. Diet also rapidly changed the composition of the indigenous microbiota, but with some taxa differentially affected between HFHSD periods. *L*. *plantarum* was not integrated into indigenous bacterial community networks according to co-occurrence patterns but still conferred distinct effects on bacterial species diversity and microbiota stability largely in a diet-dependent manner. Metagenome predictions supported the premise that *L*. *plantarum* dampens the effects of diet on the microbiome. This strain also consistently altered the predicted genetic content in the distal gut by enriching for genes encoding glyosyltransferases and bile salt hydrolases. Our findings demonstrate the interactions between ingested, transient probiotic bacteria and intestinal bacterial communities and how they can differ depending on host diet.

## Introduction

The human gastrointestinal (GI) tract is a microbial ecosystem subject to tremendous selective pressures from the host epithelium and immune and endocrine systems as well as dietary inputs comprised of a vast assortment of carbohydrates, proteins, fats, vitamins, toxins/anti-nutrients, and microorganisms. Although the indigenous bacterial colonists of the GI tract show remarkable stability, these organisms are still vulnerable to GI perturbations and “sweeps” due to external insults. Disease, antibiotics, and some medications can each result in rapid shifts to the gut microbiome as well as sustained, long-term modifications to microbial composition and function^1–3^. Similarly, alterations to the abundant macronutrients of the diet confer global changes to the intestinal community structure^4, 5^. Studies performed in rodent models have shown that the intestinal microbiota responds robustly to diet perturbations independent of host genotype and that the abundance of certain taxa can reflect previous diet exposures^6^. Maternal diets high in fat and low in microbiota-accessible carbohydrates can also result in bacterial extinctions over several generations that cannot be restored through diet alone^7^.

Less understood is the role of host diet on the GI fitness of transient bacteria consumed in foods and beverages. For foodborne pathogens, the intestinal microbiota can influence the capacity of those organisms to cause disease in a diet-dependent manner^8–10^. For example, colonization and virulence of enterohaemorrhagic *Escherichia coli* was increased in a high-fiber diet background that stimulated intestinal butyrate synthesis^11^. The importance of host diet on the fitness of commensal or probiotic bacteria in the GI tract was shown with the mono-association of *Lactobacillus plantarum* WCFS1 in germ-free mice^12^. *L*. *plantarum* colonized the GI tract in significantly higher quantities when the mice were fed a low-fat plant-polysaccharide rich diet (LFPPD) as opposed to a diet high in fat and sugar (HFHSD). The numbers of *L*. *plantarum* corresponded well to the transcriptome of this organism in the cecum of mice fed the HFHSD, which showed evidence of nutrient starvation (e.g. down-regulation of genes required for metabolism and DNA replication and up-regulation of genes for amino acid biosynthesis and transport)^12^. Remarkably, the opposite result was reached in conventionally raised mice, whereby HFHSD consumption resulted in higher numbers of *L*. *plantarum* surviving GI tract transit^13^. This occurred even though there was still evidence of nutrient starvation among *L*. *plantarum* in HFHSD-fed animals. The mice also responded differently in that colonic IL-10 levels were higher and trinitrobenzene sulfate (TNBS)-induced colitis was significantly attenuated when *L*. *plantarum* was administered in the background of the HFHSD compared to the LFPPD^13^.

A primary difference between germ-free and conventionally raised mice is the presence of an indigenous microbiome. Therefore, the survival and persistence of *L*. *plantarum* in those studies might have been due to ecological constraints resulting from a fully colonized digestive tract. In that regard, the composition of the cecal microbiota was also significantly different between mice on the LFPPD and HFHSD^13^. Among the diet-induced differences, *Lactobacillus* species were severely and significantly depleted with a HFHSD^13^. Reduced *Lactobacillus* numbers were also found for other rodent studies in which obesogenic diets were used^14, 15^. Reductions in certain *Lactobacillus* species were also similarly observed in some human dietary studies in which a high-fat, refined sugar diet was consumed^16^, whereas other studies obtained the opposite result^17, 18^. However, it remains to be determined the extent to which diet-associated, intestinal behaviors of ingested and indigenous *Lactobacillus* species are reversible and whether transient *Lactobacillus* populations are integrated within the indigenous microbiome.

**Figure 1:**
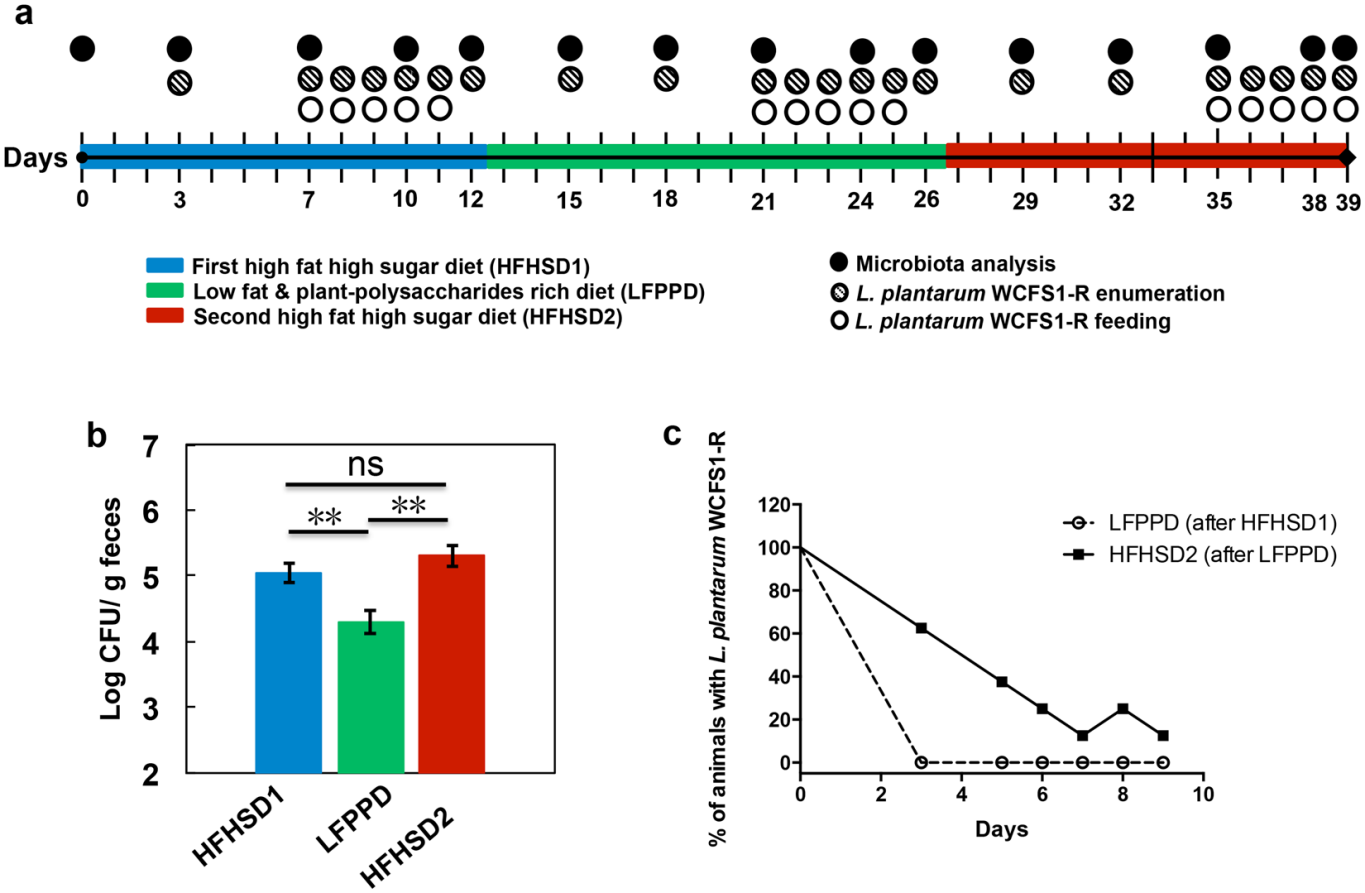
*L*. *plantarum* survived in higher numbers and persisted for longer periods in mice on HFHSD. (**a**) Experimental design of the mouse study. Mice starting on day 0 were switched from standard rodent chow to a HFHSD for 12 days (HFHSD1, blue), to a LFPPD for 14 days (green), and then back again to the HFHSD for a final 12 days (HFHSD2, red). *L*. *plantarum* WCFS1-R was fed orally during the last five consecutive days for each diet period (open circles). Mouse stools were collected at time points indicated in the figure for either *L*. *plantarum* enumeration (hatched circles) or 16S rRNA gene sequencing and qPCR (filled circles). (**b**) Culturable *L*. *plantarum* WCFS1-R in mouse stools 24 h after feeding as determined by colony enumeration. Rifampicin-resistant colonies were not detected in fecal samples collected from PBS-fed mice. The lower limit of detection was log_10_ 3.5 CFU/g stool. Data of total 8 mice for 5 consecutive days is shown. **P < 0.01, Mann-Whitney U test. (**c**) *L*. *plantarum* WCFS1-R persistence in the mouse feces after the HFHSD1 period (during LFPPD consumption) and after the LFPPD period (during HFHSD2 consumption), as determined by CFU enumeration.

In this study, we hypothesized that the open ecological niche created for ingested *L*. *plantarum* by a HFHSD is reversible and that *L*. *plantarum* interactions with the intestinal microbiome are diet dependent. To investigate these possibilities, we exposed conventionally raised mice to dietary switches between a HFHSD and LFPPD with/without the addition of *L*. *plantarum* (Fig. 1a). The survival of *L*. *plantarum* and its associations with indigenous intestinal bacteria were investigated.

## Results

### HFHSD consistently increases the survival and persistence of *L*. *plantarum* in the GI tract

Viable *L*. *plantarum* was recovered in similar numbers from all stools 3 and 5 h after the first administration of *L*. *plantarum* WCFS1-R during each of the dietary periods (Supplementary Fig. S1a). The following day and then 24 h after each of the remaining 4 days of *L*. *plantarum* feeding showed this organism survived in significantly higher numbers in the GI tract of mice fed the HFHSD compared to LFPPD (Fig. 1b). Viable *L*. *plantarum* was recovered in 10-fold greater quantities during both HFHSD periods than during the LFPPD period (Fig. 1b). These results were supported by qPCR assessments of total *L*. *plantarum* in the stools which showed an even greater increase (up to 100-fold) when the strain was administered with the HFHSD as opposed to the LFPPD (Supplementary Fig. S1b).

Diet also affected the intestinal persistence of *L*. *plantarum*. This strain was still detected in the stools of five mice (out of eight) three days after the diet switch from LFPPD to HFHSD2 (Fig. 1c). Two mice still harbored the WCFS1-R strain six days later (Fig. 1c), and this result was not due to a “cage effect”. By comparison, *L*. *plantarum* was absent from all stools collected three days after the mice were switched from the HFHSD1 to the LFPPD (Fig. 1c).

### Murine gut microbiota responds discordantly to diet switches

**Figure 2:**
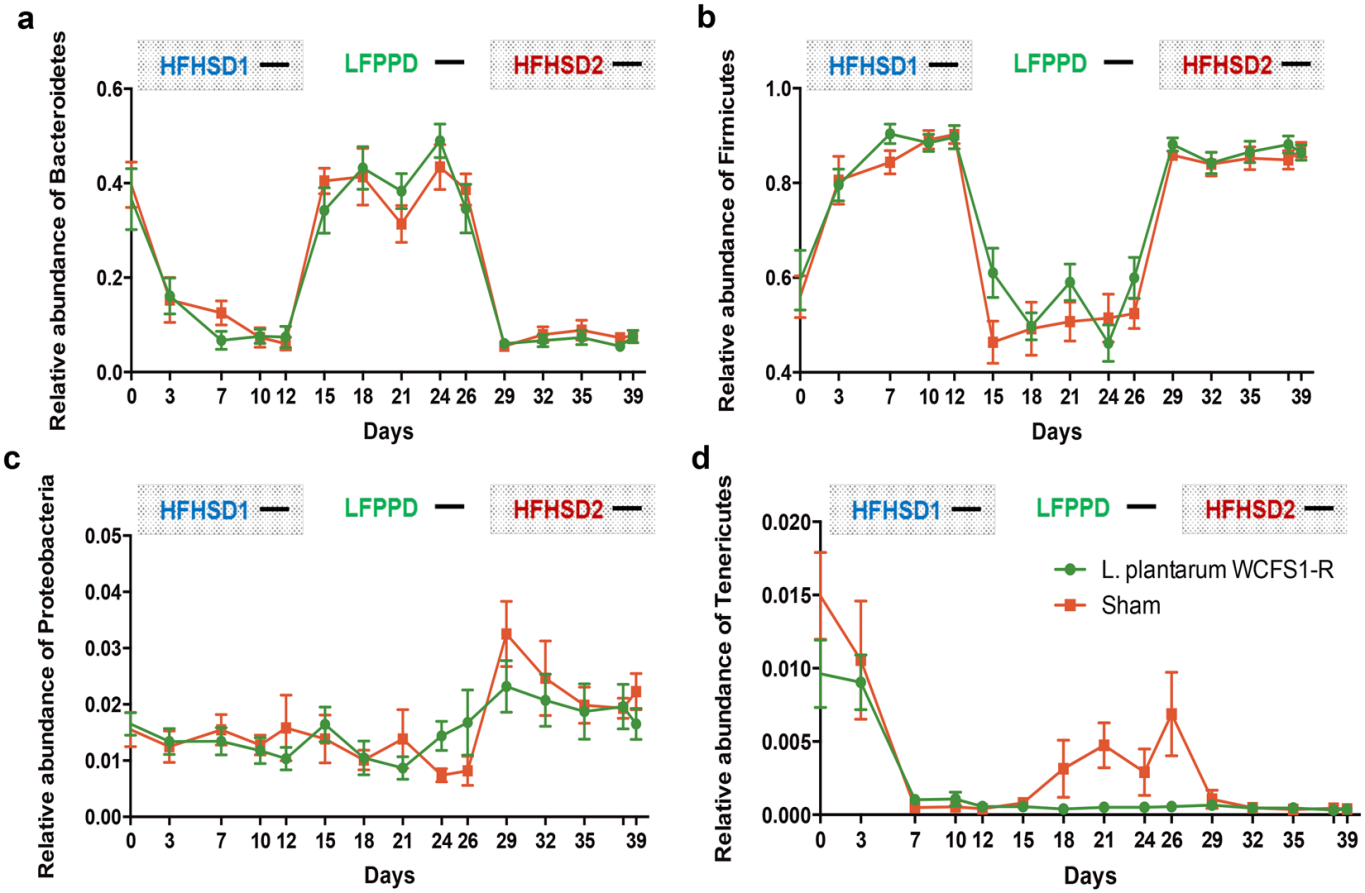
Diet switches result in significant changes to major bacterial phyla in the mouse intestine. *Bacteroidetes* (**a**), *Firmicutes* (**b**), *Proteobacteria* (**c**) and *Tenericutes* (**d**) are shown. Black dashes indicate the days when the mice received *L*. *plantarum* WCFS1-R.

The diet shifts resulted in rapid and global changes to the composition of the murine GI microbiota. Principal coordinate analysis of the weighted UniFrac matrix showed a distinct separation in the fecal bacteria according to the diet being consumed (47.7% variance, HFHSD vs. LFPPD) and in a manner that was independent of *L*. *plantarum* WCFS1-R consumption (Supplementary Fig. S2). *Bacteroidetes* reached as low as 6.5% of the total intestinal microbiota during the HFHSD periods. These quantities were significantly lower (P < 0.0001) than at the start of the study (baseline) (38.2 ± 3.9%) and during the LFPPD (41.4% ± 2.2%) (Fig. 2a). Conversely, *Firmicutes* constituted 89.4% and 86.6% of the stool microbiota during HFHSD1 and HFHSD2, respectively. These percentages were significantly higher compared to levels at the start of the study and during LFPPD consumption (P < 0.0001) (Fig. 2b).

**Figure 3:**
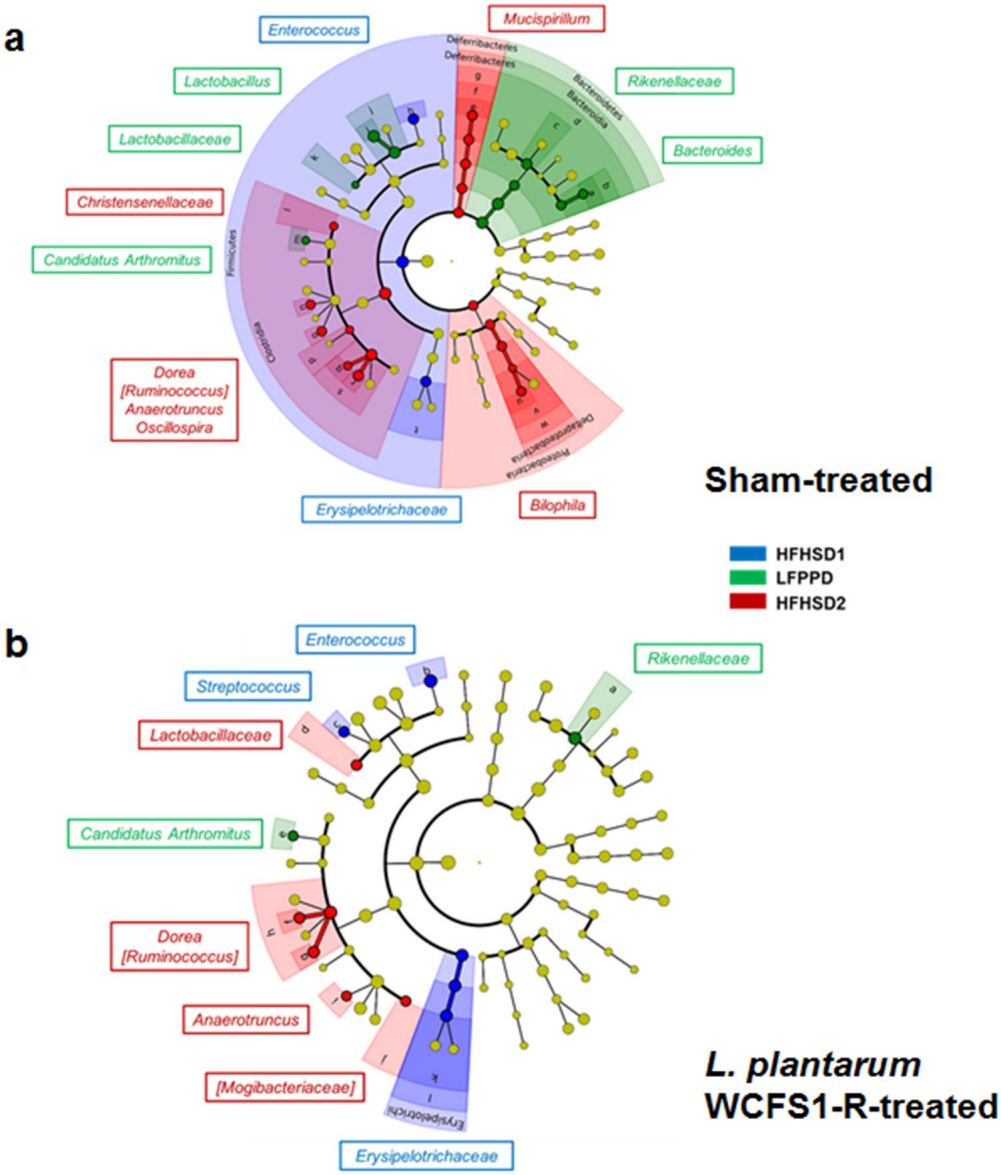
Taxonomic cladogram comparing the fecal microbiota response to different diets. Panel (**a**) and (**b**) correspond to the results from sham-treated and *L*. *plantarum* WCFS1-R fed mice respectively. Blue, green and red nodes are bacterial taxa significantly increased during the first HFHSD (HFHSD1), LFPPD and the second HFHSD (HFHSD2). Yellow nodes indicate non-significant changes in certain bacterial taxa in response to diet.

There was also evidence of discordant responses among the microbiota to the two HFHSD periods in both sham and *L*. *plantarum –* fed mice. During HFHSD1, mice harbored significantly higher proportions of the *Firmicutes* taxa *Enterococcus* and *Erysipelotrichaceae* relative to either LFPPD or HFHSD2 consumption (Fig. 3). During the HFHSD2 period, other *Firmicutes* in the *Dorea*, [*Ruminococcus*] (*Lachnospiraceae* family), and *Anaerotruncus* genera were enriched (Fig. 3) as well as *Proteobacteria* (Fig. 2c).

### *L*. *plantarum* confers modest changes to the intestinal microbiota composition

Ingesting *L*. *plantarum* WCFS1-R resulted in alterations to the intestinal microbiota that were more limited in scope compared to the introduction of a new diet. Notable, however, were the altered alpha-diversity indices during *L*. *plantarum* and LFPPD consumption. The Shannon diversity index increased (data not shown) and significantly higher numbers of observed species were detected in the *L*. *plantarum* fed mice (Supplementary Fig. S3).

The proportions of some bacterial taxa were also modified between the *L*. *plantarum* and sham-fed controls. During HFHSD1, the relative abundance of *Streptococcus* was significantly higher in mice fed *L*. *plantarum* (Fig. 3). Conversely, during the LFPPD period, sham (but not *L*. *plantarum*-fed) mice were enriched with *Tenericutes* (Fig. 2d) and *Bacteroides* (Fig. 3), the former of which was enriched in sham (control)-fed animals even prior to the introduction of *L*. *plantarum* during that dietary period. Fewer changes to the gut microbiota as a consequence of *L*. *plantarum* ingestion were also found during HFHSD2. Only the sham control mice harbored elevated levels of *Mucispirillum* (*Deferribacteres* phylum), *Christensenellaceae* and *Oscillospira* (*Firmicutes* phylum) as well as *Bilophila*, a genus of sulfite-reducing bacteria in *Proteobacteria* phylum (Fig. 3).

### Diet switches and *L*. *plantarum* alter the diversity and proportions of indigenous lactobacilli in the mouse intestine

**Figure 4:**
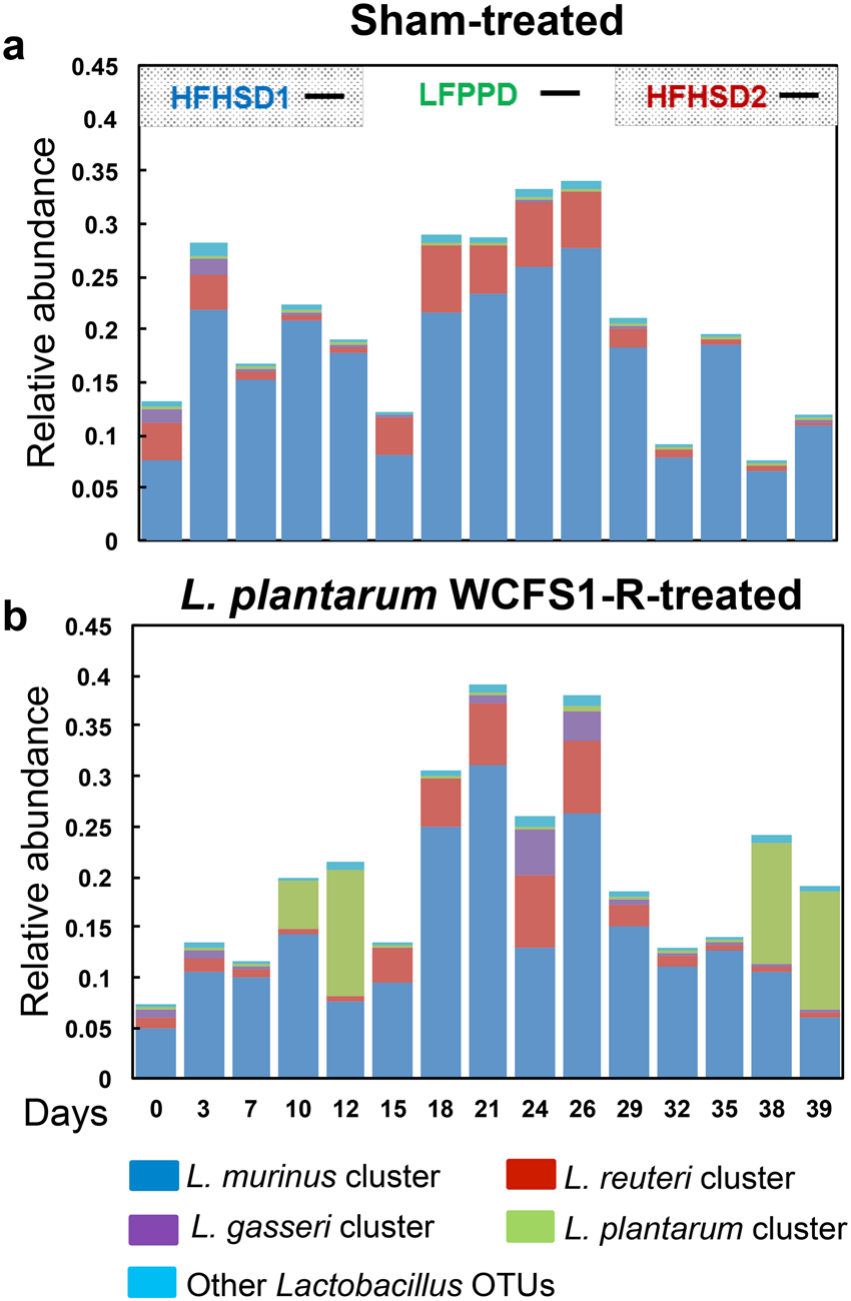
Proportions of intestinal *Lactobacillus* species changed over time during diet switches. Panel (**a**) and (**b**) correspond to the results from sham-treated and *L*. *plantarum* WCFS1-R fed mice respectively. OTUs sharing 100% sequence similarity with its nearest neighbors are shown. Average values of all 8 mice in each group were plotted. Black dashes indicate the days when the mice received *L*. *plantarum* WCFS1-R.

**Figure 5:**
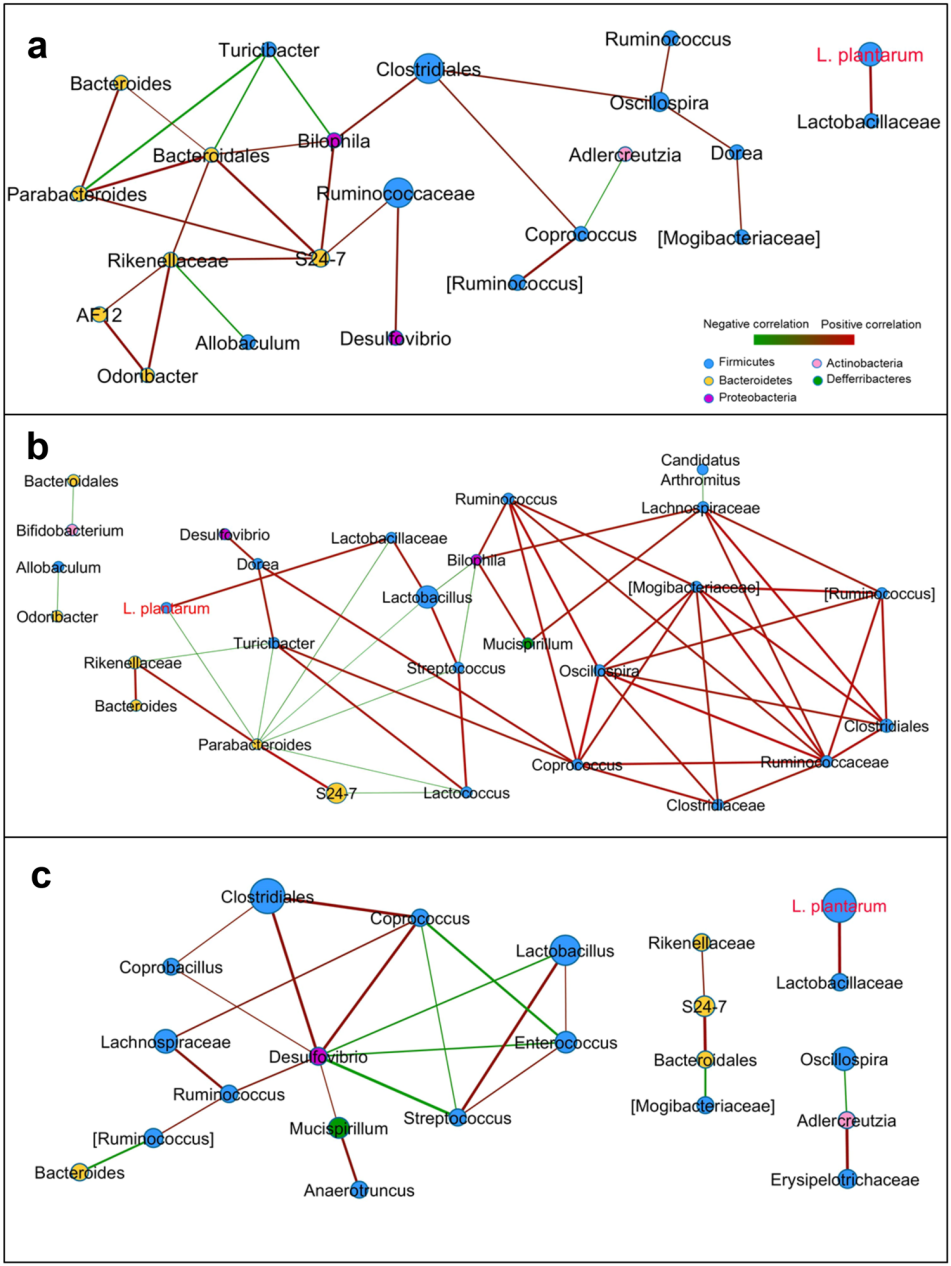
Network analysis showed various interactions among *L*. *plantarum* WCFS1-R, indigenous *Lactobacillus* and other gut microbes. Intestinal microbiota during *L*. *plantarum* WCFS1-R consumption on HFHSD1 (day 10, day 12) (**a**), LFPPD (day 24, day 26) (**b**) and HFHSD2 (day 38, day 39) (**c**) were analyzed. Each node represents one genus with the color indicating the phylum to which the genus belongs and the size of the node proportional to the relative abundance of that organism in the bacterial community (only taxa with the average abundance over 0.001 are shown). The line between nodes indicates the Spearman correlation and the color intensity indicates the correlation coefficient (red, positively correlated and green, negatively correlated). The weight of the line corresponds to the correlation significance. Taxa in brackets are based on annotations suggested by the Greengenes database.

The proportions of *Lactobacillus* in the mouse stools ranged from 7.3% to 37.6% of the total bacteria present in all mice. Opposite to *L*. *plantarum* WCFS1-R, the levels of indigenous *Lactobacillus* species (encompassing all OTUs assigned to that genus except for *L*. *plantarum*) were lowest during HFHSD consumption and increased with the LFPPD (Fig. 4). These numbers did not change with the onset of *L*. *plantarum* feeding. To this end, *L*. *plantarum* WCFS1-R did not either positively or negatively influence the levels of the indigenous *Lactobacillus* present. Instead, when *L*. *plantarum* was given to the animals during the two HFHSD periods, *L*. *plantarum* constituted a higher percentage of the total *Lactobacillus* population (41.9% and 55.9% for HFHSD1 and HFHSD2 respectively) than during the LFPPD (1.4%) (Fig. 5b).

BLAST searches of the prominent *Lactobacillus* OTUs present in the mouse stools identified *L*. *plantarum* as well as other taxa highly related to *L*. *murinus*, *L*. *reuteri*, and *L*. *gasseri* and here referred to as species clusters (Supplementary Fig. S4). Lactobacilli in the *L*. *murinus* cluster were predominant (Fig. 4 and Supplementary Fig. S5a). In the sham-fed mice, the *L*. *murinus* cluster increased from 19.3% to 26.8% and then down to 8.8% of the total intestinal bacteria as the diet changed from HFHSD1 to LFPPD and then to HFHSD2 (P = 0.1 HFHSD1 vs. LFPPD; P < 0.0001 LFPPD vs. HFHSD2) (Fig. 4a). Quantification of *L*. *murinus* cell numbers by qPCR gave similar results (Supplementary Fig. S5a). These values were also similar in mice fed *L*. *plantarum* WCFS1-R (Fig. 4b and Supplementary Fig. S5a).

Bacteria highly related to *L*. *reuteri* were the second most abundant *Lactobacillus* species cluster in the mice (Fig. 4 and Supplementary Fig. S5b). A rapid and significant 250-fold reduction in *L*. *reuteri* occurred during the first HFHSD period compared to baseline (day 0) for all mice (Supplementary Fig. S5b). Similar to *L*. *murinus*, when the animals were fed the LFPPD, the quantities of *L*. *reuteri* increased back to levels present at the start of the study (P < 0.0001 LFPPD vs. HFHSD1). The quantities declined again when mice were given the HFHSD for the second time (P < 0.0001 HFHSD2 vs. LFPPD) (Supplementary Fig. S5b).

Lastly, proportions of the *L*. *gasseri* cluster were also diet-dependent and significantly reduced with the HFHSD in all mice compared to baseline levels (P < 0.0001 baseline vs. HFHSD1) (Fig. 4 and Supplementary Fig. S5c). However, during LFPPD consumption, the quantities of the *L*. *gasseri* cluster only increased in those mice fed *L*. *plantarum* (P < 0.0005 HFHSD1 vs LFPPD) (Supplementary Fig. S5c). Once the HFHSD2 was introduced, the numbers of *L*. *gasseri* cluster declined again (P < 0.0001 LFPPD vs HFHSD2), even with *L*. *plantarum* WCFS1-R administration (Supplementary Fig. S5c).

### *L*. *plantarum* WCFS1-R has limited interactions with intestinal bacteria

We next employed network analysis to identify interactions between the mouse microbiota that changed with diet and ingestion of *L*. *plantarum*. Both diet-independent and –dependent co-occurrence patterns were found. *Clostridiales* (or *Clostridiaceae*) and *Coprococcus* were positively correlated in all dietary periods (r = 0.72, 0.76, 0.9 respectively), indicating the associations between these taxa are independent of the host diet (Fig. 5). Diet-dependent interactions included the positive correlations between *Rikenellaceae* and S24-7 (r = 0.75 and 0.7 for HFHSD1 and HFHSD2, respectively) and *Bacteroidales* and S24-7 (r = 0.83 and 0.9 for HFHSD1 and HFHSD2, respectively) occurring during both HFHSD periods (Fig. 5a and c). Co-occurrence patterns between native *Lactobacillus* species and other bacterial taxa also varied according to diet. Positive correlations were identified between *Lactobacillus* and *Streptococcus* during LFPPD and HFHSD2 (LFPPD; r = 0.78; HFHSD2, r = 0.81) and *Lactobacillus* and *Enterococcus* during HFHSD2 (r = 0.69) (Fig. 5). Conversely, negative correlations were observed between *Lactobacillus* and *Parabacteroides* during LFPPD (r = −0.83) and with *Desulfovibrio* during HFHSD2 (r = −0.71) (Fig. 5).

**Figure 6:**
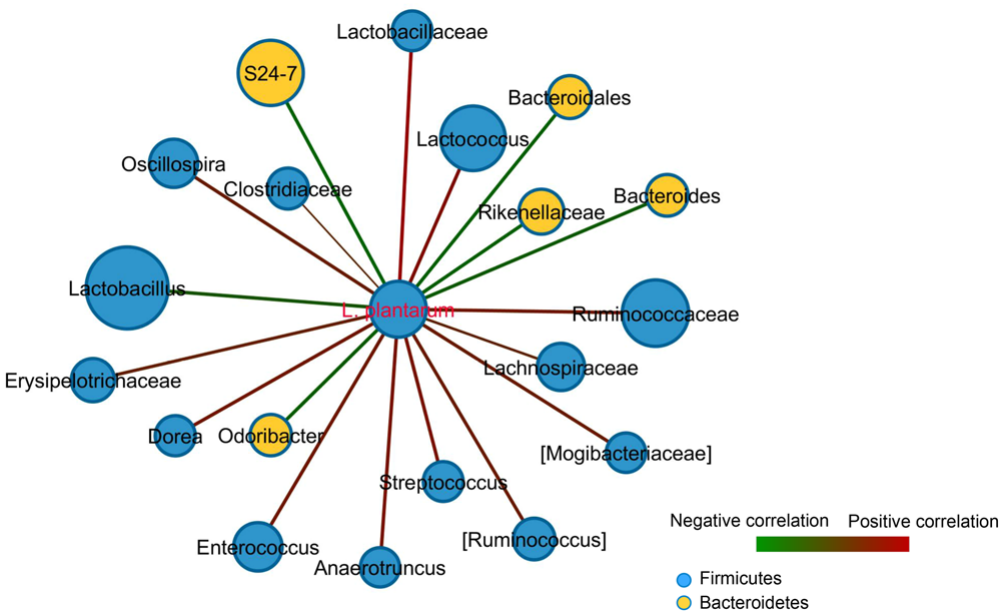
Interactions between *L*. *plantarum* and the host indigenous intestinal microbes during all three dietary periods. Each node represents one genus with the color indicating the phylum to which the genus belongs and the size of the node proportional to the relative abundance of that organism in the bacterial community (only taxa with the average abundance over 0.001 are shown). The line between nodes indicates the Spearman correlation and the color intensity indicates the correlation coefficient (red, positively correlated and green, negatively correlated). The weight of the line positively corresponds to the correlation significance. Taxa in brackets are based on annotations suggested by the Greengenes database.

Unlike indigenous lactobacilli, *L*. *plantarum* (and presumably primarily strain WCFS1) was not well integrated into the co-occurrence networks. During both HFHSD periods, *L*. *plantarum* was only associated with those bacteria classified as unknown *Lactobacillaceae* (Fig. 5a and c). We defined unknown *Lactobacillaceae* as those OTUs that could not be assigned to the genus level. Those bacteria were present at a low abundance (ranging from 0% to 1.3% of the total bacteria present). During the LFPPD consumption, *L*. *plantarum* was additionally (negatively) associated with *Parabacteroides* (r = −0.71) (Fig. 5b). This result is consistent with the overall inverse correlation between *L*. *plantarum* and members of the *Bacteroidetes* phylum when all dietary periods were compared (Fig. 6).

### Predicted bacterial gene contents change upon diet switches and *L*. *plantarum* consumption

Modifications to the indigenous microbiota with the dietary switches also resulted in significant changes in the abundance of metabolic and biosynthetic pathways in the intestinal metagenomes according to PICRUSt^19^. A total of 9 KEGG pathways including “signal transduction”, “metabolism of cofactors and vitamins”, “membrane transport”, “cellular processes and signaling” and “cell motility” were repeatedly increased in the fecal microbiota of the sham-fed controls when the HFHSD was consumed (Supplementary Fig. S6). During LFPPD consumption, genes belonging to “glycan biosynthesis and metabolism” were enriched. Most of those diet-dependent differences in predicted metagenome pathways were absent in mice fed *L*. *plantarum*.

**Figure 7:**
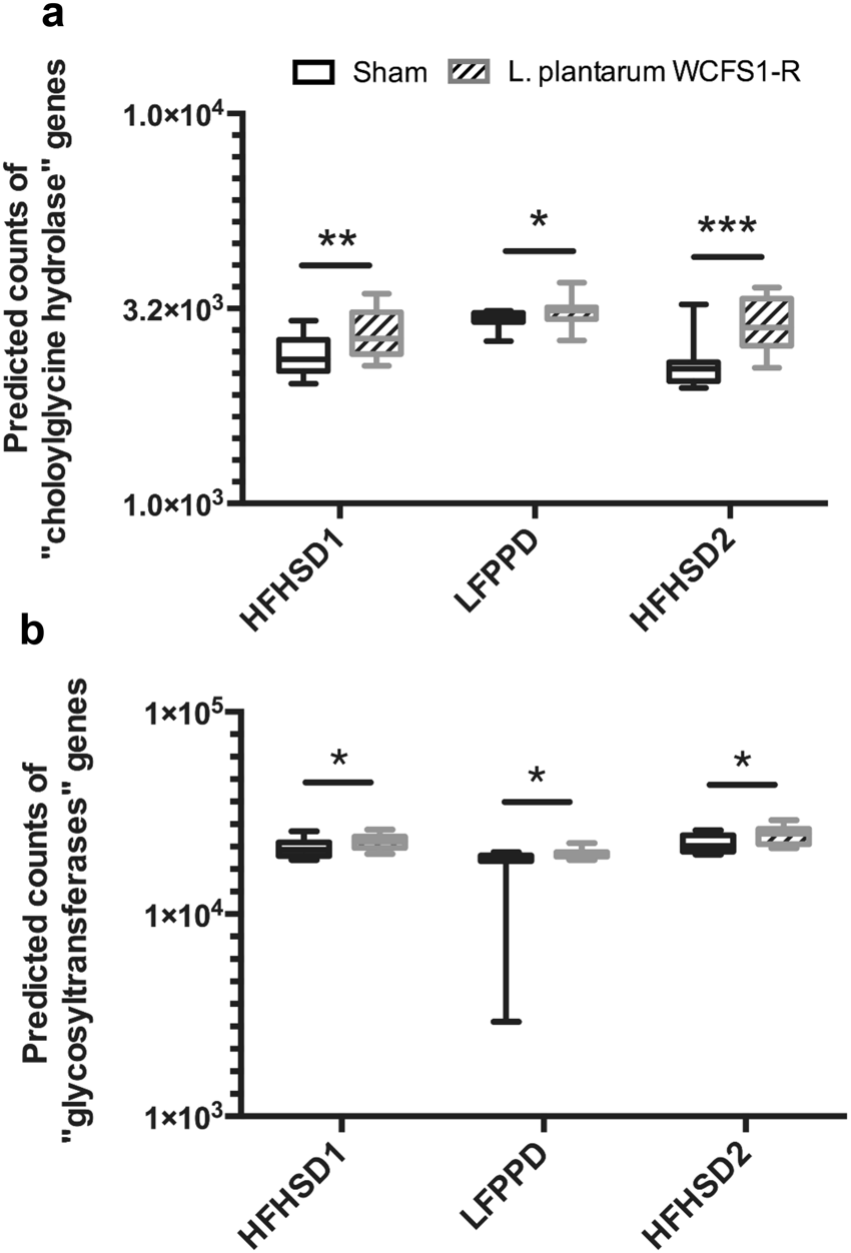
Pathways specifically enriched in *L*. *plantarum* WCFS1-R fed mice during all dietary periods. Metagenomics prediction was performed using PICRUSt^19^, a software to infer the gene content from bacterial community composition data. The whiskers of the box plot indicate the minimum and maximum value.

During each of the three dietary periods, *L*. *plantarum* ingestion instead resulted in changes to the predicted metagenomes compared to the sham-fed mice. A total of 11 KEGG pathways were enriched with *L*. *plantarum* during HFHSD1 (Supplementary Fig. S7a). Nine of those pathways were also increased during HFHSD2 and included “arachidonic acid metabolism”, “carotenoid biosynthesis” and “ion channels” (Fig. 7a and Supplementary Fig. S7). During the LFPPD period, the discriminant pathways were shifted towards genes coding for “genetic information processing” and “cellular processes” (Supplementary Fig. S7b). Lastly, *L*. *plantarum* also conferred a few diet-independent changes to the intestinal microbiome. *L*. *plantarum* fed mice were enriched for genes coding for bile acid metabolism (choloylglycine hydrolase) and glycosyltransferases across all dietary periods (Fig. 7b). Further analysis showed that most predicted reads of the choloylglycine hydrolase gene (K01442) originated from lactobacilli (Supplementary Table S3).

## Discussion

Because ingested probiotic bacteria typically reside in the GI tract for only short periods of time (days to weeks), their initial interactions with the epithelium and indigenous microbiota are pivotal to determining the overall significance of these microorganisms to human health. Our results show that host diet dramatically alters the survival and persistence of ingested *L*. *plantarum* and that indigenous *Lactobacillus* species are oppositely and repeatedly impacted. Ingested *L*. *plantarum* WCFS1-R therefore might fill a vacant ecological niche left by the HFHSD-induced reductions to indigenous *Lactobacillus* species. Unlike those species, however, *L*. *plantarum* was not integrated into the microbial co-occurrence networks and therefore the indigenous microbiome. Instead, *L*. *plantarum* exerted independent, and likely more transient, effects on the intestinal community structure and function.

*L*. *plantarum* WCFS1-R survived the GI tract in 10- to 100-fold higher numbers in the current and previous studies^13^ when the mice were fed the HFHSD. Herein, the results were verified by three methods (colony enumeration, 16S rRNA DNA sequencing, and qPCR) and were in agreement with the higher levels of intestinal persistence when administration was stopped. There are several potential reasons why the numbers of *L*. *plantarum* were greater in the HFHS dietary background. One possibility is that nutritional preferences of *L*. *plantarum* are more suited to the HFHSD when in the presence of an indigenous microbiota. The importance of nutritional resources in the intestine was previously shown for *E*. *coli*, whereby the capacity to consume distinct sugars prevented nutritional-niche overlap and enabled co-colonization of different commensal *E*. *coli* strains^20^. The primary carbohydrate in the HFHSD used in this study is sucrose. Although *L*. *plantarum* WCFS1-R is able to consume sucrose, it is unlikely that sucrose utilization conferred a nutritional advantage to *L*. *plantarum* because sucrose is rapidly absorbed in the small intestine^21^. To this regard, we were unable to detect sucrose in the stools of mice fed the same HFHSD in a different study (data not shown).

Global restructuring of the indigenous microbiota in response to dietary shifts is another factor that could explain *L*. *plantarum* GI fitness. Consistent with numerous other studies in humans and mice, we found that the host diet is a significant determinant of the gut microbiome. Diet-induced changes can even overrule host genotype and inter-personal variation^6, 22^. Within three days after the switch to the HFHSD, the intestinal microbiota composition was altered to the extent that the ratio of the dominant phyla *Firmicutes* and *Bacteroidetes* was significantly increased as shown in some human studies^23–25^. Several of the HFHSD-enriched taxa (e.g. *Erysipelotrichaceae*, *Enterococcus*, and *Bilophila*) were also present in greater proportions in obese humans^4, 26, 27^. The predicted metagenomes of the sham-fed mice indicate that bacteria enriched with HFHSD possess higher numbers of genes for “xenobiotics degradation”, “lipid metabolism”, and “cell motility”. By comparison, LFPPD enriched for bacterial taxa specialized for “glycan biosynthesis and metabolism”. This finding is consistent with prior metagenome analyses of diet-induced changes to the murine gut microbiota^28^. However, it is notable that there were some differences between the two HFHSD periods. Changes in certain taxa and predicted metagenome pathways between HFHSD1 and HFHSD2 were indicative of recently reported hysteresis effects in the microbiota observed following serial dietary switches^6^.

Predominant and repeatable changes with HFHSD were reductions to the quantities of indigenous *Lactobacillus* species. The absolute (by qPCR) and relative (by DNA sequencing) numbers of each *Lactobacillus* species cluster were lower during both HFHSD periods. The abundant species were highly related to *L*. *murinus*, *L*. *gasseri*, and *L*. *reuteri*, lactobacilli recognized to be indigenous inhabitants of rodent intestines^29, 30^ and the human GI tract^31^. Reduced quantities of these bacteria during HFHSD2 confirmed that losses to indigenous *Lactobacillus* cell numbers were not due to an initial response of the mouse to the altered diet, but rather caused by robust, ecosystem-level changes in the intestine.

Therefore, increases in *L*. *plantarum* survival and persistence during HFHSD consumption could be largely the consequence of an open ecological niche in the intestine left by reductions to the indigenous *Lactobacillus* species. In other words, survival of *L*. *plantarum* might have been supported within the HFHSD but not LFPPD backgrounds due to reduced competition with indigenous *Lactobacillus* species. This possibility is in agreement with prior findings showing that dietary *Lactobacillus* survives longer in ampicillin-treated mice containing no detectable indigenous lactobacilli^32^. The intestinal persistence of *Lactococcus lactis* CNCM I-1631 in both animal models and human subjects was also positively associated with a resident gut microbiota containing a low prevalence of closely related species^33^. Similarly, prolonged persistence of *Bifidobacterium longum* AH1206 in human subjects was correlated to initially low levels of resident *B*. *longum* and an under-representation of carbohydrate utilization genes in the resident GI tract microbiota^34^.

However, this competitive exclusion hypothesis apparently does not apply to all ingested bacteria. Instead, there appears to be circumstances or certain functional relationships that fit the “like will to like” rule observed for some enteric pathogens and *L*. *reuteri* ^35^. Although we did not find such a relationship between *L*. *plantarum* and the indigenous lactobacilli, the quantities of *L*. *plantarum* were strongly and positively correlated with other members of the *Firmicutes* phylum, including other lactic acid bacteria such as *Streptococcus* (Fig. 6). Moreover, it is notable that during the LFPPD period the *L*. *gasseri* cluster was only enriched on the days in which WCFS1-R was consumed. This finding is consistent with a prior study in which *Lactobacillus* species diversity increased in mice fed *L*. *plantarum* and *L*. *casei* in combination with a regular rodent chow diet^36^. Such associations indicate that certain *Lactobacillus* species/strains might be compatible in the intestine and could be confirmed in studies in which different *Lactobacillus* strains are deliberately paired to evaluate co-colonization potential based on predicted cooperative or antagonistic cell traits.

Pivotal to predicting *Lactobacillus* survival and persistence is understanding the diet-induced, selective pressures in the intestine. *Lactobacillus* can be grouped as either host- or environment- associated^37, 38^. Host-associated species include *L*. *murinus*, *L*. *gasseri*, *L*. *johnsonii*, and *L*. *reuteri*; whereas *L*. *plantarum*, an organism that is essential to numerous food fermentations, is regarded to have a broader distribution^39^. A difference between these species is that unlike host-associated lactobacilli, *L*. *plantarum* and other broad-host range *Lactobacillus* species tend to harbor more genes dedicated to oxidative stress tolerance^38, 40, 41^. This distinction was supported by the larger numbers of genes required for tolerance to reactive oxygen species (ROS) in the genome of *L*. *plantarum* WCFS1 compared to the genomes of representative strains of *L*. *murinus*, *L*. *reuteri* and *L*. *gasseri* (Supplementary Table S4). Resistance to ROS might be an important ecological fitness determinant in the intestine during exposure to obesogenic diets. It is understood that HFHSD consumption elevates ROS concentrations at localized sites in the GI tract^15, 42^. Such altered intestinal redox conditions can result in the enrichment of aero-tolerant bacteria at the expense of oxygen-sensitive populations^43^, including some indigenous *Lactobacillus* species^15, 42^. Therefore, ROS-tolerant *L*. *plantarum* WCFS1-R might have benefited in the HFHSD background by gaining access to otherwise limited resources previously utilized by indigenous lactobacilli.

Beyond the compensatory changes to *Lactobacillus* numbers, ingestion of *L*. *plantarum* WCFS1-R appears to have also exerted independent effects on the composition of the intestinal microbiota. Overall, *L*. *plantarum* conferred a stabilizing influence and there were fewer significantly-changing, diet-responsive taxa in mice fed *L*. *plantarum* WCFS1-R. This result is in agreement with other studies indicating that ingested *Lactobacillus* could serve to maintain gut microbiota stability in individuals with intestinal diseases, allergies, or on antibiotics^44–46^. For example, mice consuming *L*. *plantarum* WCFS1-R did not harbor significantly increased proportions of *Bilophila*, a pro-inflammatory pathobiont enriched in diets high in saturated fat^4, 47^. *L*. *plantarum* WCFS1-R was also negatively correlated with *Parabacteroides* during the LFPPD period. *Parabacteroides* levels were elevated in patients with colorectal cancer^48^ and in elderly people in long-term residential care compared to the community-dwelling elders and young healthy subjects^49^. Lastly, it is notable that the proportions of the low-abundant phylum *Tenericutes* increased in sham-fed animals with LFPPD in a manner that was independent of *L*. *plantarum* exposure within that dietary period. It is possible that this difference between the sham- and *L*. *plantarum*- fed groups was due to the previous contact of those animals with *L*. *plantarum* WCFS1 during HFHSD1.

Mice fed *L*. *plantarum* also contained altered metagenomes according to predictions with PICRUSt. *L*. *plantarum* WCFS1-R resulted in significant changes to multiple KEGG pathways, some in a diet-dependent manner. Moreover, there were multiple, diet-associated KEGG pathways that were no longer changed within the indigenous microbiota upon *L*. *plantarum* WCFS1-R consumption. Independent of host diet, *L*. *plantarum* consistently enriched bile acid metabolism genes and glycosyltransferase genes. This could be caused by the introduction of the WCFS1-R strain, the genome of which contains multiple copies of bile salt hydrolase and glycosyltransferase genes^50, 51^. Lastly, during the LFPPD period, even though *L*. *plantarum* constituted a small percentage of the bacteria present in the distal gut, mice fed *L*. *plantarum* WCFS1-R were predicted to contain increases in numbers of genes coding for “cell motility” and “carbohydrate metabolism”. These genes were also enriched in human studies upon probiotic consumption, suggesting that there are certain core responses to exogenous *Lactobacillus* by the host microbiome^52, 53^. Because the accuracy of PICRUSt is based on the availability and annotation of known bacterial genomes, other methods such as metagenomics and metabolomics are needed to confirm and further understand the functional significance of *L*. *plantarum in vivo*.

The intestinal ecosystem is remarkably resistant to exogenous microorganisms^54, 55^. Commensal or probiotic bacteria usually result in minor disturbances in the distal gut in human adult populations^56^. Such changes might not be trivial, however, because we showed that even though *L*. *plantarum* WCFS1-R was not well integrated within the indigenous microbiota, ingestion of this strain did have consequences on the gut microbiota structure in a diet-dependent manner. The high-fat and high- sugar diet diminished the indigenous *Lactobacillus* population while supported *L*. *plantarum* survival in the mouse model tested here, suggesting the consumption of exogenous *Lactobacillus* species might provide compensatory effects on the microbiota which are augmented in obesogenic Western diets. This is of special interest because *Lactobacillus* is increasingly recognized to have pivotal roles in host development, immune tolerance, and drug sensitivity^57–60^. Provision of exogenous (probiotic) *Lactobacillus* strains, with appropriate stress-tolerance, nutritional, and effector capacities, could be usefully applied to replenish lactobacilli lost due to the consumption of obesogenic diets. Alternatively, symbiotic approaches might be needed for probiotic *Lactobacillus* when administered under unfavorable dietary (or other) conditions.

## Methods

### *L*. *plantarum* WCFS1-R preparation and enumeration

*L*. *plantarum* WCFS1 is a single colony isolate of *L*. *plantarum* NCIMB 8826, a strain able to survive in the human GI tract^61^. A spontaneous rifampicin-resistant mutant of *L*. *plantarum* WCFS1 (WCFS1-R) was isolated and used throughout the study as previously described^13^. For each day of oral administration, *L*. *plantarum* was grown in de Mann Rogosa and Sharpe (MRS) medium (BD, Franklin Lakes, NJ, USA) until exponential phase was reached (an optical density (OD) at 600 nm of approximately 0.5). The cells were collected by centrifugation at 4,000 rpm for 10 min, washed twice with phosphate-buffered saline (PBS) (137 mM NaCl, 2.7 mM KCl, 10 mM Na_2_HPO_4_, 2 mM KH_2_PO_4_, pH 7.4), and then suspended in a volume of 20 μl PBS (approximately 10^9^ cells) for mice to drink off of the tip of a gavage bulb following procedures described in Lee *et al*.^62^. This method provides a quantitative and reproducible compromise between controlled invasive orogastric gavage and provision of the strain in drinking water. Enumeration of rifampicin-resistant *L*. *plantarum* from mouse feces was performed as previously described^62^. Briefly, feces were diluted with PBS, homogenized by mixing with 2 mm-glass beads for 10 sec at 4 m/sec twice in 1 min intervals in a Fast Prep 24 instrument (MP Biomedicals, Solon, OH, USA). Serial dilutions of the fecal suspensions were then plated onto MRS agars containing 50 μg ml^−1^ Rifampicin (Thermo Fisher Scientific, Waltham, MA, USA) and incubated at 37 °C for two days prior to enumeration.

### Mouse study design

All procedures were performed under the protocol approved by the UC Davis Animal Care and Use Committee (protocol # 17899). A total of 16 female BALB/c mice (Harlan, Livermore, CA, USA) were housed four per cage and given free access to food and water on a 12 h light/dark cycle. During the first 5 days, mice were fed the 2018 Teklad Global 18% Protein Rodent Diet (Harlan, Livermore, CA, USA), a standard rodent chow that is enriched in plant-polysaccharides. Animals then received a high fat and high refined sugar diet (HFHSD) (Research Diet D12079B, Research Diet, New Brunswick, NJ, USA) for 12 days (HFHSD1, Fig. 1a). Next, the mice were switched onto a low- fat and plant-polysaccharide rich diet (LFPPD) (Teklad 2014 Global 14% Protein Rodent Maintenance Diet, Harlan, Livermore, CA, USA) for the subsequent 14 days. Lastly, mice were switched back to the HFHSD for 12 days prior to sacrifice (HFHSD2). Fat and sucrose contributed 43% and 41% of the energy content of the HFHSD respectively (Supplementary Table S1), and for the LFPPD, 13% energy was from fat and 67% energy from carbohydrates, mainly consisting of plant-derived polysaccharides (Supplementary Table S2).

For comparisons to *L*. *plantarum* feeding, eight (out of 16) mice consumed 10^9^ *L*. *plantarum* WCFS1-R per day for the last 5 days during each diet period and the remaining animals (n = 8) were sham fed with PBS in the same volume as used to deliver the bacterial culture (Fig. 1a). Freshly expelled stools were examined for culturable *L*. *plantarum* prior to *L*. *plantarum* administration, at 0 h, 3 h and 5 h after the first feeding, and then every 24 h for the remainder of the dietary period (Fig. 1a). Fresh stools were also collected at five different time points during each diet, flash frozen in liquid nitrogen, and stored at −80 °C for gut microbiota analysis (Fig. 1a). The body weights of all animals were measured every two to three days for the duration of the study; however, no differences in body weights were found between the sham and *L*. *plantarum* fed mice (data not shown).

### 16S rRNA gene sequencing and data analysis

Total bacterial DNA was extracted from 240 frozen mouse stool samples and the V4 region of community 16S rRNA genes were amplified by PCR as previously described^63^. Pooled PCR amplicons were sequenced according to the paired-end protocol on the Illumina MiSeq platform (Illumina Inc., San Diego, CA, USA) at the UC Davis Genome Center (http://dnatech.genomecenter.ucdavis.edu/).

Raw fastq files from both ends were assembled, demultiplexed and analyzed in the QIIME 1.8.0 (Quantitative Insights Into Microbial Ecology) software package^64^. An average of 25,876 high quality reads were obtained for each sample, and 544 Operational Taxonomic Units (OTUs) with an abundance over 0.005% of the total reads were identified with 97% similarity using a closed reference OTU picking method. Representative sequences from each OTU were assigned to their corresponding taxonomy based on the GreenGenes database (version 13_8)^65^. Beta diversity was calculated using the UniFrac distance between samples^66^ and visualized based on principle coordinate analysis (PCoA). Linear discriminant analysis (LDA) effect size analysis (LEfSe)^67^ was used to identify discriminant bacterial species for each diet with/without *L*. *plantarum* feeding. Pair-wise Spearman correlation with FDR (Benjamini & Hochberg method) adjusted P value was performed for bacterial network analysis. Correlation networks were visualized in an open source software platform Cytoscape (version 3.2.1)^68^. Bacterial gene contents were predicted through Phylogenetic Investigation of Communities by Reconstruction of Unobserved States (PICRUSt, version 1.0.0)^19^. Discriminant gene categories were identified using LEfSe^67^. When indicated, discriminant genes were further filtered through the fold-change analysis with a cut-off of over a 2-fold change to be considered significant. The DNA sequences were deposited in the Sequence Read Archive (SRA) (http://www.ncbi.nlm.nih.gov/sra) with the accession number SRP057487.

*Lactobacillus* species were identified by blasting OTU sequences to NCBI 16S rRNA gene database (http://blast.ncbi.nlm.nih.gov). A neighbor-joining phylogenetic tree was constructed in MEGA6^69^ with *Lactobacillus* and *Lactobacillaceae* OTU sequences and 16S rRNA gene sequences of their representative closest neighbor downloaded from NCBI (Supplementary Fig. S4). Due to the high sequence similarity of 16S rRNA gene V4 region among *Lactobacillus* species, OTUs were classified to species clusters, composed of *Lactobacillus* with identical V4 regions. The clusters were designated as follows: *Lactobacillus murinus* cluster includes *L*. *murinus*, *Lactobacillus faecis*, *Lactobacillus apodemi* and *Lactobacillus animalis*; *Lactobacillus reuteri* cluster includes *L*. *reuteri*, *Lactobacillus antri*, *Lactobacillus frumenti*, *Lactobacillus panis* and *Lactobacillus oris*; *Lactobacillus gasseri* cluster includes *L*. *gasseri*, *Lactobacillus hominis*, *Lactobacillus johnsonii* and *Lactobacillus taiwanensis*; *Lactobacillus plantarum* cluster includes *L*. *plantarum*, *Lactobacillus fabifermentans*, *Lactobacillus paraplantarum* and *Lactobacillus pentosus*.

### *Lactobacillus* species-specific, quantitative PCR

Genomic DNA of *L*. *plantarum* WCFS1-R, *Lactobacillus murinus* ASF 361, *Lactobacillus reuteri* ATCC 23272 and *Lactobacillus gasseri* ATCC 19992 were extracted using the DNeasy Blood & Tissue Kit (Qiagen Inc., Valencia, CA, USA), quantified with the Quant-iT™ PicoGreen® dsDNA Assay Kit (Life Technologies, Carlsbad, CA, USA). The DNA was then serially diluted and used to construct standard curves ranging from 10 to 10^7^ of 16S rRNA gene copies per reaction for absolute quantification. A total of five 16S rRNA genes for each *L*. *plantarum* genome and six 16S rRNA genes for each *L*. *murinus*, *L*. *reuteri* and *L*. *gasseri* genome^70, 71^ was used to estimate corresponding *Lactobacillus* cell numbers and 16S rRNA gene copy numbers in fecal samples.

*L*. *plantarum*, *L*. *murinus*, *L*. *reuteri* and *L*. *gasseri* - specific PCR primer sequences were either selected from the literature^72–75^ or designed using Primer 3^76^ (Table 1). These primers were also selective for closely related *Lactobacillus* species according to Primer-BLAST^77^. Primers for *L*. *reuteri* were found to be distinct for members of the *L*. *reuteri* species. Primers for *L*. *plantarum* were predicted to also target *L*. *pentosus*. Those for *L*. *murinus* were specific for *L*. *murinus*, *L*. *animalis* and *L*. *crispatus*, and for *L*. *gasseri*, the primers were limited to *L*. *gasseri*, *L*. *johnsonii*, *L*. *taiwanensis* and *L*. *hominis*. Real-time, quantitative PCR (qPCR) was performed in an ABI 7500 Fast Real-time PCR system (Applied Biosystems, Carlsbad, CA, USA). Each reaction contained SsoFast EvaGreen supermix with low ROX (2X) (Bio-Rad, Hercules, CA, USA), 250 nM of each primer (Life Technologies, Carlsbad, CA, USA) and approximately 30 ng of fecal bacterial genomic DNA. PCR amplification was initiated at 98 °C for 3 min, followed by 40 cycles of 98 °C for 5 sec and either 60 °C for 30 sec for total bacteria, *L*. *plantarum* and *L*. *murinus*, 57 °C for 30 sec for *L*. *reuteri*, and 60 °C for 45 sec for *L*. *gasseri*. Each reaction was performed in duplicate. A melting curve was added at the final stage to confirm the amplification specificity. PCR amplification efficiency was calculated each time based on the standard curve and data were only considered to be valid when the amplification efficiency was within 90–100%.

**Table 1:** *Lactobacillus* species-specific real-time PCR primers used in the study.

Targets	Primer1	Primer2	Sequence_Primer1 (5′–>3′)	Sequence_Primer2 (5′–>3′)	Tm	Amplicon size (bp)	References
16S rRNA	UniF	UniR	GTGSTGCAYGGYYGTCGTCA	ACGTCRTCCMCNCCTTCCTC	53.5/55.8	~128	Belenguer, A. *et al*.^72^
*L*. *plantarum* 16S rRNA	lplan_vreg1_F	lplan_vreg1_R	TTACATTTGAGTGAGTGGCGAACT	AGGTGTTATCCCCCGCTTCT	62.2/62.1	71	Klocke, M. *et al*.^73^
*L*. *gasseri* 16S rRNA	LactoF	LgassR	TGGAAACAGRTGCTAATACCG	CAGTTACTACCTCTATCTTTCTTCACTAC	62/60	322	Byun, R. *et al*.^74^
*L*. *reuteri* 16S rRNA	LreutF	LreutR	GCTTGCACCTGATTGACGAT	AGCCATGTGGCTTTTGTTGT	61.2/60.6	134	This study
*L*. *murinus* 16SrRNA	LmurF	LmurR	TCGAACGAAACTTCTTTATCACC	CGTTCGCCACTCAACTCTTT	60/60	63	Bindels, L. B. *et al*.^75^

### Statistical analysis

Statistical analyses and plots were generated in Graphpad Prism 5.0 (GraphPad Software, Inc., La Jolla, CA, USA). For the heat map image of the metagenomics prediction, R studio (Version 0.98.1091, RStudio, Inc., Boston, MA, USA) was used. Unless specified, non-parametric statistical comparisons were performed, including Mann-Whitney U test and Kruskal-Wallis one-way analysis of variance with Dunn’s post hoc tests.

## Electronic supplementary material

Supplementary Information
