# Supplementary material for: Dietary perturbations alter the ecological significance of ingested Lactobacillus plantarum in the digestive tract
Source: Sci Rep. 2017 Aug 4;7:7267. doi: 10.1038/s41598-017-07428-w (PMC5544775; doi:10.1038/s41598-017-07428-w)
Supplement: Supplementary file 1 — Supplementary Information [file 41598_2017_7428_MOESM1_ESM.doc]

**Dietary perturbations alter the ecological significance of ingested *Lactobacillus* *plantarum* in the digestive tract**

Xiaochen Yin^1^, Bokyung Lee^1,2^, Jose Zaragoza^1,3^ and Maria L. Marco^1^

^1^ Department of Food Science and Technology, University of California, Davis, USA

Correspondence:

Maria L. Marco

Department of Food Science & Technology

One Shields Avenue

University of California, Davis

Davis, CA 95616

Email: mmarco@ucdavis.edu

Phone: 530-574-4893

^2^ Current address: Center for Comparative Medicine, Department of Anatomy, Physiology and Cell Biology, School of Veterinary Medicine, University of California, Davis, CA

^3^ Current address: Bayer Crop Science, West Sacramento, CA

**Supplementary Information**


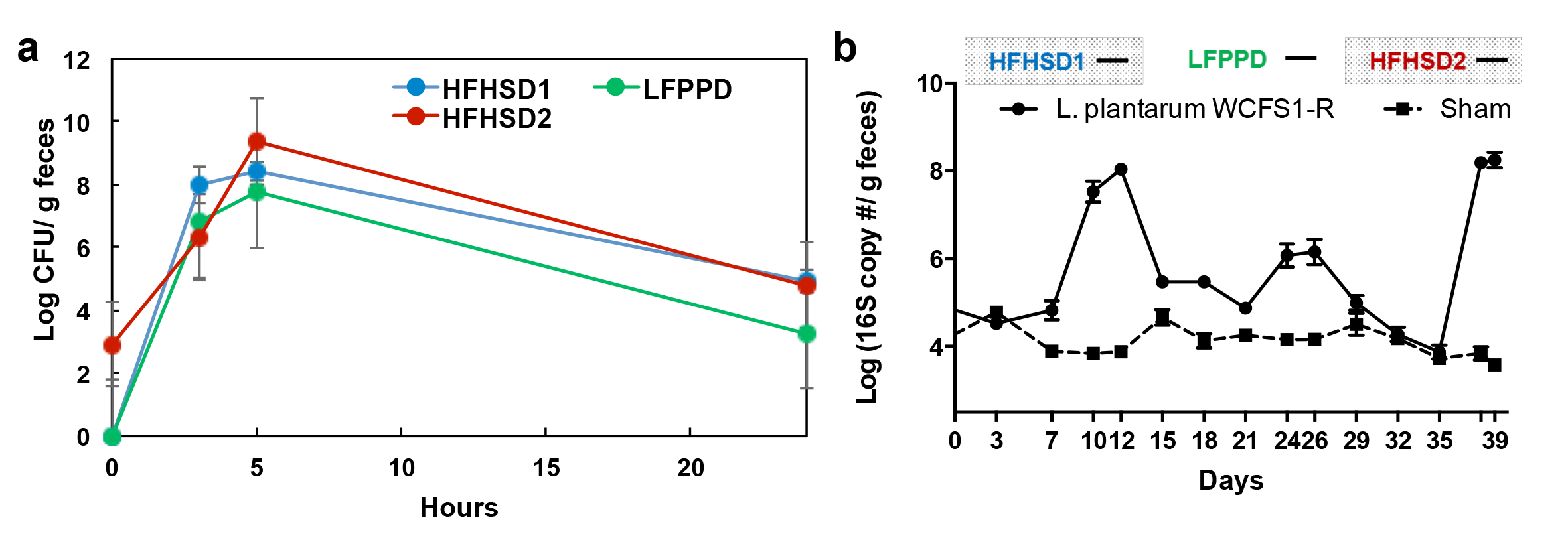


**Supplementary Fig. S1. *L. plantarum* survived better and persisted longer in the murine gut during HFHSD.** (a) *L. plantarum* WCFS1-R cultured from the stools over the course of the first day of *L. plantarum* WCFS1-R feeding for each diet. (b) *L. plantarum* qPCR quantification for sham and *L. plantarum* WCFS1-R fed animals during each HFHSD and LFPRD period. The black bars on top indicate the periods when PBS or *L. plantarum* WCFS1-R was fed. For both (a) and (b), values for 8 mice at each time point are shown as avg ± ste.


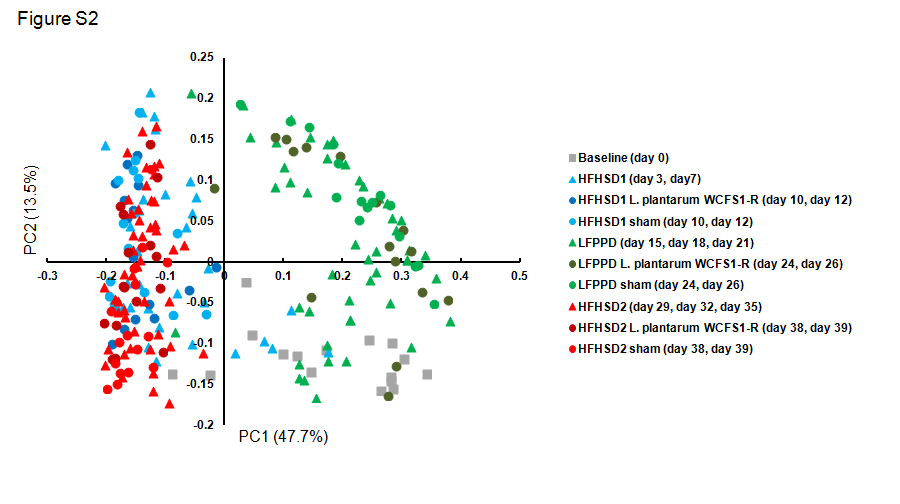


**Supplementary Fig. S2. Consistent and repeatable effects of HFHSD on the intestinal microbiota of mice.** Weighted UniFrac PCoA of the intestinal bacterial microbiota is shown.


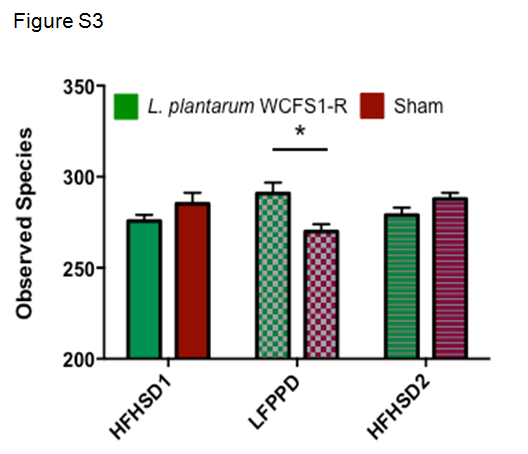


**Supplementary Fig. S3. Observed number of species for *L. plantarum* feeding and sham control groups during each dietary period.** Specifically, samples from day 10, day 12 for HFHSD1, day 24, day 26 for LFPPD and day 38, day 39 for HFHSD2 were used for the calculation. * P<0.05 as calculated from Mann-Whitney U test.


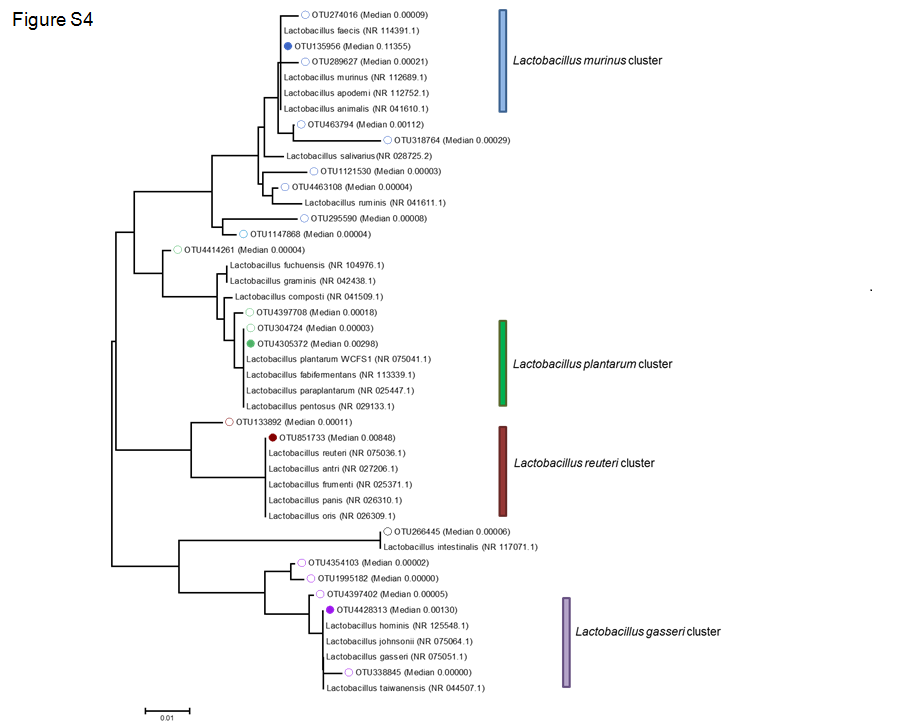


**Supplementary Fig. S4. Phylogenetic analysis based on sequences of *Lactobacillus* and *Lactobacillaceae* OTUs and their nearest neighbors.** *Lactobacillus* OTUs with 100% identity to the nearest neighbors were shown in filled circles with its median abundance included in parentheses (OTU 135956, 851733, 4428313 and 4305372). *Lactobacillaceae* (OTU 4414261, 4397708 and 304724) and the rest *Lactobacillus* OTUs are indicated with empty circles and the median abundance in parentheses. Representative sequences of *Lactobacillus* species were used and the accession numbers were included in parentheses.


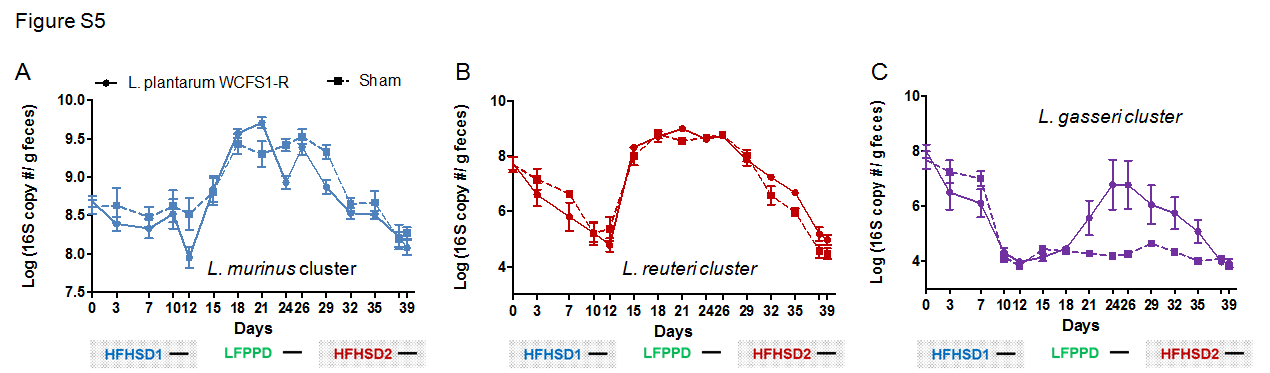


**Supplementary Fig. S5. Indigenous *Lactobacillus* species clusters responded differently to diet switches and *L. plantarum* feeding according to real-time qPCR.** Quantities of *L. murinus* cluster (a)*, L. reuteri* cluster (b)*,* and *L. gasseri* cluster (c) are shown for both sham (squares) and *L. plantarum* WCFS1-R (circles) fed mice. Values are avg ± ste of 8 mice at each time point. Black dashes indicate the days when the mice received *L. plantarum* WCFS1-R.


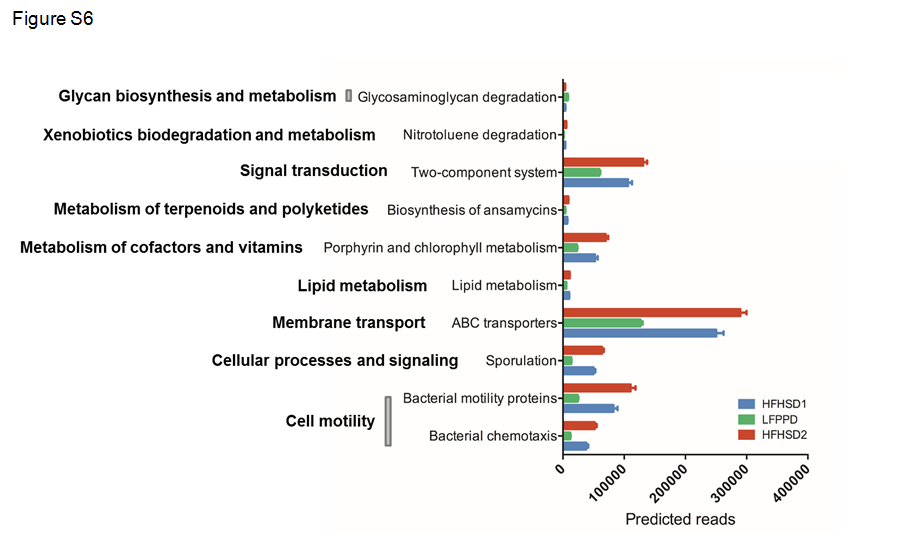


**Supplementary Fig. S6. Significantly changed gene categories in response to dietary switches. Bacterial gene contents were predicted using PICRUSt** [**^1^**](#_ENREF_1)**.** Discriminant categories (identified by LEfSe [^2^](#_ENREF_2)) with greater than a 2- fold change in abundance are shown.


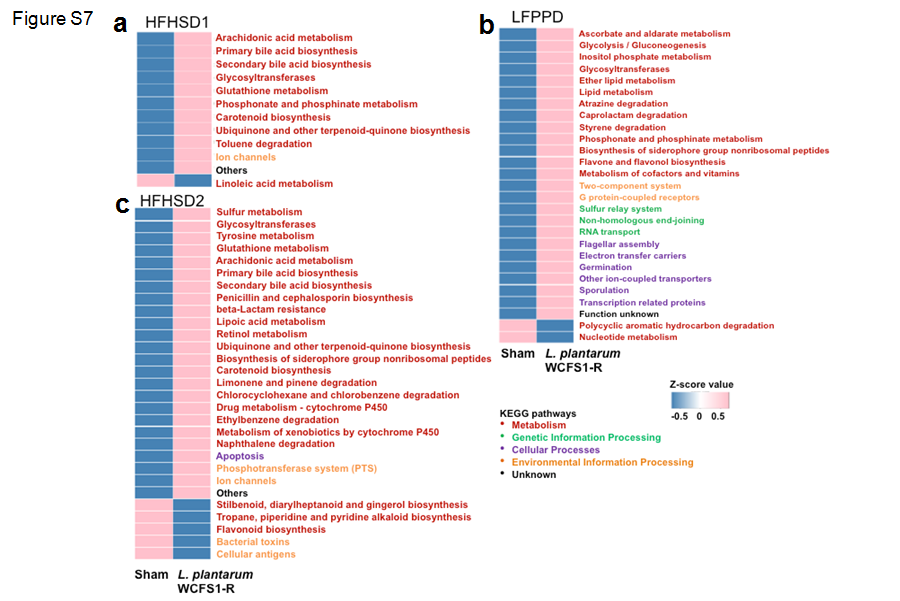


**Supplementary Fig. S7. Significantly changed KEGG pathways in response to *L. plantarum* feeding during HFHSD1 (a), LFPPD (b) and HFHSD2 (c).** Bacterial gene contents were predicted using PICRUSt [^1^](#_ENREF_1). Discriminant categories identified by LEfSe [^2^](#_ENREF_2) are shown.

Supplementary Table S1. The composition of high-fat, high-sugar (HFHSD) diet.

| **HFHS diet, Research Diet, Product #D12079B** | | |
| --- | --- | --- |
| **Energy Density** | **kcal/g** | **4.7** |
| Calories from protein | % | 17 |
| Calories from fat | % | 43 |
| Calories from carbohydrate | % | 41 |
| **Ingredient** | **g/kg** | **kcal** |
| Casein | 195 | 780 |
| DL-methionine | 3 | 12 |
| Corn starch | 50 | 200 |
| Maltodextrin 10 | 100 | 400 |
| Sucrose | 341 | 1364 |
| Cellulose | 50 | 0 |
| Anhydrous milk fat | 200 | 1800 |
| Corn oil | 10 | 90 |
| Mineral mix S10001 | 35 | 0 |
| Calcium carbonate | 4 | 0 |
| Vitamin mix V10001 | 10 | 40 |
| Choline bitartrate | 2 | 0 |
| Cholesterol | 1.5 | 0 |
| Ethoxyquin | 0.04 | 0 |
| **Total** | **1001.54** | **4686** |

Supplementary Table S2. The composition of low-fat, plant-polysaccharides rich diet (LFPPD).

| **LFPP diet, Teklad global 14% protein rodent maintenance diet (Harlan Laboratories)** | | |
| --- | --- | --- |
|
| **Energy Density** | **kcal/g** | **2.9** |
| Calories from protein | % | 20 |
| Calories from fat | % | 13 |
| Calories from carbohydrate | % | 67 |
| **Macronutrients** | **percent weight** |  |
| Crude protein | % | 14.3 |
| Fat | % | 4 |
| Carbohydrate (available) | % | 48 |
| Crude fiber | % | 4.1 |
| Neutral detergent fiber | % | 18 |
| Ash | % | 4.7 |
| **Ingredients (in descending order of inclusion):** | |  |
| Wheat middlings, ground wheat, ground corn, corn gluten meal, calcium carbonate, soybean oil,dicalcium phosphate, iodized salt, L-lysine, vitamin E acetate, DL-methionine,magnesium oxide, choline chloride, manganous oxide, ferrous sulfate, menadione sodium bisulfite complex (source of vitamin K activity), zinc oxide, copper sulfate, niacin, calcium pantothenate, calcium iodate, pyridoxine hydrochloride, riboflavin, thiamin mononitrate, vitamin A acetate, vitamin B12 supplement, folic acid, cobalt carbonate, biotin, vitamin D3 supplement. | | |
|
|
|
|
|

**Supplementary Table S3. OTUs contributing over 5% to the overall predicted reads of the bile salt hydrolase gene (K01442).**

| **Fecal samples** | **OTU ID** | **Contribution to the reads of this group** | **Representative nearest neighbour (BLAST)** | **Identity** | **Accession number** |
| --- | --- | --- | --- | --- | --- |
| **HFHSD1 + *L. plantarum* WCFS1-R** | 4305372 | 0.26 | *Lactobacillus plantarum* WCFS1 | 100% | KC429782.1 |
|  | 1100972 | 0.16 | *Lactococcus lactis* subsp. *cremoris* NBRC 100676 | 100% | NR_113925.1 |
|  | 135956 | 0.11 | *Lactobacillus murinus* NBRC 14221 | 100% | NR_112689.1 |
|  | 351309 | 0.10 | *Acetatifactor muris* CT-m2 | 93% | NR_117905.1 |
|  | 1033413 | 0.06 | *Enterococcus hirae* LMG 6399 | 100% | NR_114783.2 |
| **HFHSD1 + PBS** | 135956 | 0.22 | *Lactobacillus murinus* NBRC 14221 | 100% | NR_112689.1 |
|  | 1100972 | 0.17 | *Lactococcus lactis* subsp. *cremoris* NBRC 100676 | 100% | NR_113925.1 |
|  | 351309 | 0.11 | *Acetatifactor muris* CT-m2 | 93% | NR_117905.1 |
|  | 1033413 | 0.07 | *Enterococcus hirae* LMG 6399 | 100% | NR_114783.2 |
| **LFPPD + *L. plantarum* WCFS1-R** | 851733 | 0.25 | *Lactobacillus reuteri* DSM 20016 | 100% | NR_075036.1 |
|  | 135956 | 0.17 | *Lactobacillus murinus* NBRC 14221 | 100% | NR_112689.1 |
|  | 4428313 | 0.06 | *Lactobacillus gasseri* ATCC 33323 | 100% | NR_075051.1 |
|  | 242029 | 0.05 | *Muribaculum intestinale* YL27 | 96% | NR_144616.1 |
| **LFPPD + PBS** | 135956 | 0.23 | *Lactobacillus murinus* NBRC 14221 | 100% | NR_112689.1 |
|  | 851733 | 0.20 | *Lactobacillus reuteri* DSM 20016 | 100% | NR_075036.1 |
| **HFHSD2 + *L. plantarum* WCFS1-R** | 4305372 | 0.36 | *Lactobacillus plantarum* WCFS1 | 100% | KC429782.1 |
|  | 1100972 | 0.14 | *Lactococcus lactis* subsp. *cremoris* NBRC 100676 | 100% | NR_113925.1 |
|  | 135956 | 0.08 | *Lactobacillus murinus* NBRC 14221 | 100% | NR_112689.1 |
|  | 351309 | 0.08 | *Acetatifactor muris* CT-m2 | 93% | NR_117905.1 |
| **HFHSD2 + PBS** | 1100972 | 0.18 | *Lactococcus lactis* subsp. *cremoris* NBRC 100676 | 100% | NR_113925.1 |
|  | 351309 | 0.15 | *Acetatifactor muris* CT-m2 | 93% | NR_117905.1 |
|  | 135956 | 0.12 | *Lactobacillus murinus* NBRC 14221 | 100% | NR_112689.1 |

**Supplementary Table S4. Comparative genomics of selected *Lactobacillus* species.**

| **Representative Strain** | | | |
| --- | --- | --- | --- |
| ***L. plantarum* WCFS1** | | | |
| **Enzymes** | **Present/ Absent** | **Locus Tag (NCBI)** | **Encoded protein** |
| Superoxide dismutase | Absent | / | / |
| Catalase | Present | lp_3578 | Catalase |
| Manganese uptake | Present | lp_0275 | Manganese transport protein MntH, Nramp superfamily |
|  |  | lp_1095 | Manganese ABC transporter, ATP-binding protein |
|  |  | lp_1096 | Manganese ABC transporter, permease protein |
|  |  | lp_1097 | Manganese/zinc ABC transporter, substrate binding protein |
|  |  | lp_1295 | Manganese transport protein MntH, Nramp superfamily |
|  |  | lp_1919 | Cadmium-/manganese-transporting P-type ATPase |
|  |  | lp_2992 | Manganese transport protein |
| NADH oxidase | Present | lp_0760 | NADH oxidase |
|  |  | lp_0766 | NADH oxidase |
|  |  | lp_1925 | NADH oxidase |
|  |  | lp_1941 | NADH oxidase |
|  |  | lp_3449 | NADH oxidase |
|  |  | pWCFS103_16 | NADH oxidase (plasmid) (putative) |
| Pyruvate oxidase | Present | lp_0849 | Pyruvate oxidase |
|  |  | lp_0852 | Pyruvate oxidase |
|  |  | lp_2629 | Pyruvate oxidase |
|  |  | lp_3587 | Pyruvate oxidase |
|  |  | lp_3589 | Pyruvate oxidase |
| Peroxidase | Present | lp_0220 | Glutathione peroxidase |
|  |  | lp_1445 | NADH peroxidase |
|  |  | lp_2323 | Thiol peroxidase |
|  |  | lp_2544 | NADH peroxidase |
|  |  | lp_3430 | Peroxidase |
| Glutathione reductase | Present | lp_0369 | Glutathione reductase |
|  |  | lp_1253 | Glutathione reductase |
|  |  | lp_1822 | Glutathione reductase |
|  |  | lp_3267 | Glutathione reductase |
| Thioredoxin:thioredoxin reductase | Present | lp_0236 | Thioredoxin |
|  |  | lp_2270 | Thioredoxin |
|  |  | lp_3437 | Thioredoxin |
|  |  | lp_1351 | Thioredoxin-fold protein, DsbA family,FrnE-like subfamily |
|  |  | lp_2633 | Thioredoxin H-type |
|  |  | lp_0761 | Thioredoxin reductase |

**Supplementary Table S4. Comparative genomics of selected *Lactobacillus* species (continued).**

| **Representative Strain** | | | |
| --- | --- | --- | --- |
| ***L. murinus* ASF361** | | | |
| **Enzymes** | **Present/ Absent** | **Locus Tag (NCBI)** | **Encoded protein** |
| Superoxide dismutase | Absent | / | / |
| Catalase | Absent | / | / |
| Manganese uptake | Present | C822_RS07925 | Zinc/manganese transport system ATP-binding protein |
| NADH oxidase | Absent | / | / |
| Pyruvate oxidase | Absent | / | / |
| Peroxidase | Present | C822_RS07975 | Dyp-type peroxidase |
| Glutathione reductase | Absent | / | / |
| Thioredoxin:thioredoxin reductase | Present | C822_RS04880 | Thioredoxin |
|  |  | C822_RS06625 | Thioredoxin |
|  |  | C822_RS08690 | Thioredoxin |
|  |  | C822_RS06630 | Thioredoxin-disulfide reductase |
|  |  | C822_RS02985 | Thioredoxin-disulfide reductase |

**Supplementary Table S4. Comparative genomics of selected *Lactobacillus* species (continued).**

| **Representative Strain** | | | |
| --- | --- | --- | --- |
| ***L. reuteri* DSM 20016** | | | |
| **Enzymes** | **Present/ Absent** | **Locus Tag (NCBI)** | **Encoded protein** |
| Superoxide dismutase | Absent | / | / |
| Catalase | Absent | / | / |
| Manganese uptake | Present | LREU_RS08155 | Manganese transporter |
|  |  | LREU_RS09685 | Manganese transporter |
| NADH oxidase | Present | LREU_RS00345 | NADH oxidase |
|  |  | LREU_RS08480 | NADH oxidase, partial |
| Pyruvate oxidase | Absent | / | / |
| Peroxidase | Present | LREU_RS08120 | Peroxidase |
| Glutathione reductase | Absent | / | / |
| Thioredoxin:thioredoxin reductase | Present | LREU_RS06555 | Thioredoxin |
|  |  | LREU_RS02805 | Thioredoxin |
|  |  | LREU_RS01965 | Thioredoxin reductase |
|  |  | LREU_RS05630 | Thioredoxin reductase |

**Supplementary Table S4. Comparative genomics of selected *Lactobacillus* species (continued).**

| **Representative Strain** | | | |
| --- | --- | --- | --- |
| ***L. gasseri* ATCC33323** | | | |
| **Enzymes** | **Present/ Absent** | **Locus Tag (NCBI)** | **Encoded protein** |
| Superoxide dismutase | Absent | / | / |
| Catalase | Absent | / | / |
| Manganese uptake | Absent | / | / |
| NADH oxidase | Absent | / | / |
| Pyruvate oxidase | Present | LGAS_RS09315 | Pyruvate oxidase |
| Peroxidase | Present | LGAS_RS08940 | Cytochrome C551 peroxidase |
| Glutathione reductase | Absent | / | / |
| Thioredoxin:thioredoxin reductase | Present | LGAS_RS02095 | Thioredoxin |
| Thioredoxin:thioredoxin reductase | Present | LGAS_RS07050 | Thioredoxin |
|  |  | LGAS_RS06505 | Thioredoxin reductase |
|  |  |  |  |

**References**

1 Langille, M. G. I. *et al.* Predictive functional profiling of microbial communities using 16S rRNA marker gene sequences. *Nat Biotech* **31**, 814-821, doi:10.1038/nbt.2676 (2013).

2 Segata, N. *et al.* Metagenomic biomarker discovery and explanation. *Genome Biol* **12**, R60, doi: 10.1186/gb-2011-12-6-r60 (2011).
